# The Immediately Releasable Pool of Mouse Chromaffin Cell Vesicles Is Coupled to P/Q-Type Calcium Channels via the Synaptic Protein Interaction Site

**DOI:** 10.1371/journal.pone.0054846

**Published:** 2013-01-30

**Authors:** Yanina D. Álvarez, Ana Verónica Belingheri, Andrés E. Perez Bay, Scott E. Javis, H. William Tedford, Gerald Zamponi, Fernando D. Marengo

**Affiliations:** 1 Laboratorio de Fisiología y Biología Molecular, Instituto de Fisiología, Biología Molecular y Neurociencias (CONICET), Departamento de Fisiología y Biología Molecular y Celular, Facultad de Ciencias Exactas y Naturales, Universidad de Buenos Aires, Buenos Aires, Argentina; 2 Department of Physiology and Pharmacology, Hotchkiss Brain Institute, University of Calgary, Calgary, Canada; The University of Queensland, Australia

## Abstract

It is generally accepted that the immediately releasable pool is a group of readily releasable vesicles that are closely associated with voltage dependent Ca^2+^ channels. We have previously shown that exocytosis of this pool is specifically coupled to P/Q Ca^2+^ current. Accordingly, in the present work we found that the Ca^2+^ current flowing through P/Q-type Ca^2+^ channels is 8 times more effective at inducing exocytosis in response to short stimuli than the current carried by L-type channels. To investigate the mechanism that underlies the coupling between the immediately releasable pool and P/Q-type channels we transiently expressed in mouse chromaffin cells peptides corresponding to the synaptic protein interaction site of Cav2.2 to competitively uncouple P/Q-type channels from the secretory vesicle release complex. This treatment reduced the efficiency of Ca^2+^ current to induce exocytosis to similar values as direct inhibition of P/Q-type channels via ω-agatoxin-IVA. In addition, the same treatment markedly reduced immediately releasable pool exocytosis, but did not affect the exocytosis provoked by sustained electric or high K^+^ stimulation. Together, our results indicate that the synaptic protein interaction site is a crucial factor for the establishment of the functional coupling between immediately releasable pool vesicles and P/Q-type Ca^2+^ channels.

## Introduction

The efficiency of transmitter release in neurons and neuroendocrine cells is highly dependent on the localization of vesicles with respect to the Ca^2+^ source [1]–[3]. In chromaffin cells, the activation of voltage dependent Ca^2+^ channels (VDCCs) by short depolarizations induces the fast exocytosis of a small group of vesicles that represents 10–25% of the readily releasable pool (RRP) and is termed the immediately releasable pool (IRP) [2], [4]–[6]. Classically, the IRP was defined as a subset of readily releasable vesicles, located in close proximity of VDCCs [5]. These vesicles will thus sense a higher Ca^2+^ concentration than the rest of the RRP [4]–[7].

Chromaffin cells express different VDCCs subtypes (L, P/Q, N and R), and all of them participate in dense core vesicle exocytosis when these cells are stimulated with a train of depolarizations, or long steady depolarizations [8], [9]. It was proposed that the colocalization between IRP vesicles and VDCCs might result from a random distribution of RRP vesicles and channels [7], [10]. In this scenario, all VDCC subtypes present in chromaffin cells are expected to participate in the IRP release in proportion to their contribution to the whole voltage dependent Ca^2+^ current [9]. However, there is evidence suggesting that the different VDCC subtypes expressed in chromaffin cells are not equally efficacious in triggering exocytosis [11]–[13]. In addition, such a random distribution cannot explain the biphasic time course of exocytosis during a train of short depolarizations [14]. Alternatively, it is possible that a particular Ca^2+^ channel subtype is specifically coupled to IRP vesicles. In a recent study, our research group obtained strong evidence indicating that P/Q Ca^2+^ channels are the primary channels responsible for IRP release [15]. Particularly, we have shown that IRP exocytosis was severely inhibited (i) by the addition of ω-agatoxin IVA, a P/Q channel specific toxin, and (ii) in chromaffin cells obtained from P/Q channel knockout mouse. In contrast, when we completely blocked the L Ca^2+^ channels, which drive the biggest proportion of voltage activated Ca^2+^ currents in these cells, IRP exocytosis was not affected at all.

A specific coupling between IRP vesicles and P/Q-type channels suggest the existence of a physical interaction between these two entities at the molecular level. The α_1_ subunit of P/Q-type, as well as N-type, Ca^2+^ channels has an intracellular linker region connecting domains II and III, which is known to interact with proteins of the exocytic machinery such as syntaxin, SNAP-25 and synaptotagmin [16], [17]. There is clear evidence indicating that this region, known as the synaptic protein interaction site (synprint), maintains close physical coupling between vesicles and channels, thus improving the stimulus-secretion response in synaptic terminals [18], [19]. Therefore, it is possible that a similar type of interaction between dense core vesicles and Ca^2+^ channels may occur for the IRP in chromaffin cells. Previous results that show the feasibility of this hypothesis include the identification of synprint in different splice variants of the P/Q α_1A_ subunit in bovine chromaffin cells; the co-immunoprecipitation of P/Q channels and the SNARE complex with a monoclonal antibody against SNAP-25; and the co-localization of α_1A_ and SNAP-25 at the membrane of intact chromaffin cells [20].

In the present work we studied the role of the synprint linker region in the functional coupling between P/Q-type Ca^2+^ current and the exocytosis of vesicles associated to IRP. Our results show that the addition of exogenous synprint (to disrupt the normal interaction between the P/Q Ca^2+^ channel and exocytic/vesicular proteins) provoked a significant reduction in IRP exocytosis, without affecting calcium entry. This treatment provoked a similar effect on IRP exocytosis and on the exocytosis efficiency for short depolarizations as the pharmacological inhibition of P/Q-type Ca^2+^ current. We propose that the synprint site of α_1A_ subunit participates in the establishment of a physical interaction between P/Q calcium channels and secretory vesicles, which would be responsible of highly coupled IRP exocytosis in chromaffin cells. To our knowledge, the present work shows for the first time that the synprint sequence is crucial for highly coupled IRP exocytosis in native chromaffin cells.

## Materials and Methods

### Mouse Adrenal Chromaffin Cell Preparation

All animal procedures were performed under protocols approved by the Consejo Nacional de Investigaciones Científicas y Técnicas (Argentina), and are in accordance with the National Institute of Health Guide for the Care and Use of Laboratory Animals (NIH publication 80-23/96), USA, and local regulations. All efforts were made to minimize animal suffering and to reduce the number of animals used.

Adrenal glands from two 13–18 days old mice were used in each culture. Animals were anesthetized with an overdose of avertine, and the glands were isolated following the procedures described by Perez Bay et al [21]. Both glands were placed into a dish containing Hanks solution, and cortexes were removed mechanically. Adrenal medullas were digested for 25 min in Hanks solution containing papaine (0.5–1 mg/ml) at 37°C. Subsequently, the medullas were disrupted with a yellow tip in Dulbecco’s modified Eagle’s medium (DMEM) low glucose, supplemented with 5% fetal calf serum, 5 µl/ml peniciline/estreptomicine, 1.3 µl/ml gentamicine, 1 mg/ml bovine seroalbumin, and 10 µM citosine-1-β-D-arabinofuranoside. The cell suspension was diluted with additional medium to a final volume of 600 µl and filtered sequentially through 200 µm and 50 µm pore meshes. Cells were cultured on small pieces of poly-L-lysine pretreated coverslips, at 37°C, 95% O_2_-5% CO_2_.

Transfection procedure: Three hours after finishing the cell culture, DMEM was replaced by OPTIMEM medium (Gibco, Invitrogen Corporation. Carlsbad, CA), and the cells were transfected alternatively with a *synprint*-pIRES2-EGFP or pIRES2-EGFP plasmid (Clontech Laboratories, Takara Bio, USA) using lipofectamine 2000 (Invitrogen). After 20 min, the mixture OPTIMEM-lipofectamine-plasmid was replaced again by DMEM, ending the transfection procedure. The cells were used for experiments 24–48 hr later. The transfection treatment provoked, on average, a reduction of Ca^2+^ current and exocytosis. However, transfected cells did not show modifications in the relative contributions of P/Q- and L-type Ca^2+^ currents to the total current (see results).

### Whole Cell Patch-clamp and Membrane Capacitance Measurements

The patch-clamp set up comprised a patch-clamp amplifier (Model EPC7, LIST-MEDICAL, D-611 Darmstadt 13. Germany), a data acquisition interface (DigiData 1200 series, Axon Instruments Inc., Foster City, CA) and a personal computer. Chromaffin cells were washed in extracellular solution composed of (in mM) 120 NaCl, 20 Hepes, 4 MgCl_2_, 5 CaCl_2_, 5 mg/ml glucose and 1 µM tetrodotoxin (pH 7.3), and mounted on an inverted microscope equipped with a 100 W mercury lamp and appropriate filters to visualize EGFP fluorescence. The standard internal solution used in the patch-clamp pipettes (3–5 MΩ) contained (in mM) 95 Cs d-glutamate, 23 Hepes, 30 CsCl, 8 NaCl, 1 MgCl_2_, 2 Mg-ATP, 0.3 GTP and 0.5 Cs- EGTA (pH 7.2). These solutions were designed to selectively measure voltage dependent Ca^2+^ currents (I_Ca2+_). The holding potentials were not corrected for junction potentials. We considered that the recorded cells were “leaky”, and discarded, when the leak current measured at the normal holding potential of −80 mV was bigger than −30 pA. The cell membrane capacitance (C_m_) was measured with a software phase-sensitive detector (jClamp, Sci Soft, Branford, CT, USA). The command voltage applied to the cell was composed of the sum of a sinusoidal voltage (390 Hz, 80 mV peak to peak) and a holding potential of −80 mV. The membrane current was measured at two phase angles, *I*_φ+0_ and *I*_φ+90_, relative to the applied sinusoidal potential [22]. The output at *I*_φ+0_ represents changes in the real part of the cell admittance, and the output at *I*_φ+90_ reflect changes in the imaginary part of the cell admittance, from which we can determine the changes in membrane capacitance. The data were filtered at 3 kHz. The experiments were carried out at room temperature (22–24°C). P/Q Ca^2+^ channels were blocked by adding the specific toxin ω-agatoxin-IVA (200 nM) to the extracellular solution, and L Ca^2+^ channels were blocked with 10 µM nitrendipine.

### Estimation of Immediately Releasable Pool

**Figure 1:**
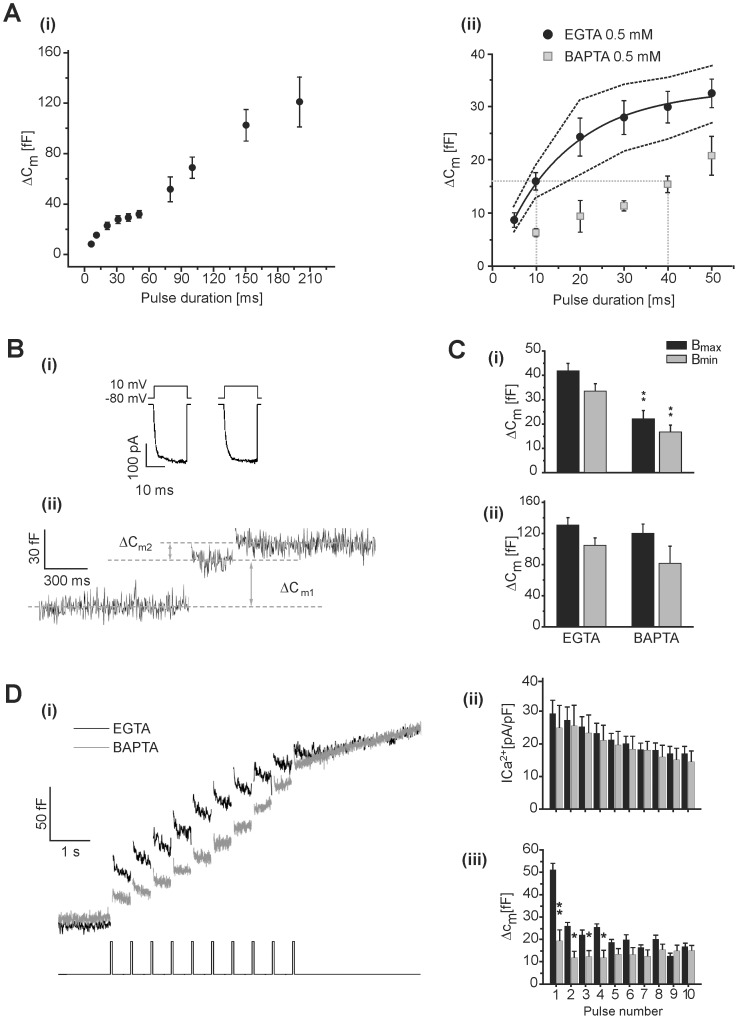
IRP exocytosis is tightly coupled to Ca^2+^ source. A. (i) Summary of capacitance increases after stimulation with depolarizing pulses of different lengths, between 5 and 200 ms, in control condition (with EGTA 0.5 mM in the internal solution). Note that there is a clearly defined initial component, which saturates approximately between 30 and 50 ms pulses. (ii) The black circles in the figure on the right is an expanded representation of this initial component (n = 116), while the gray squares represent experiments performed with BAPTA (0.5 mM) as exogenous internal buffer (n = 53). The results obtained with EGTA were fitted with a single exponential function of the form A. (1−e^−t/τ^) (A = 33±1 fF, τ = 15±0.8 ms, R = 0.998) which is represented by the continuous black line. The black dashed lines represent the confidence intervals (95%). **B.** The paired pulse protocol, composed of two 10 ms depolarization pulses separated by a 300 ms interval, which was used to calculate the IRP. The figure represents (i) the Ca^2+^ currents and (ii) the membrane capacitance changes provoked by application of this protocol during one typical experiment. The two pulses (represented in (i), from −80 to +10 mV) induced identical calcium currents, while the C_m_ response exhibited a clear depression between the first (ΔC_m1_) and the second pulse (ΔC_m2_). The values of B_min_ and B_max_ for this particular experiment were 42 and 48 fF, respectively. **C.** (i) The bar diagram summarizes the B_min_ and B_max_ values for the IRP obtained in presence of BAPTA (n = 14) or EGTA (n = 13) in the internal solution. BAPTA reduced significantly the exocytosis of IRP (p<0.02) respect to EGTA. (ii) On the other hand, the exocytosis obtained in response to a stronger stimulus (two 100 ms pulses separated by 300 ms), aimed to estimate the whole RRP [5], [25], was not affected by the type of calcium buffer added to the internal solution (EGTA, n = 11; BAPTA, n = 12). **D.** (i) Averaged exocytic response of chromaffin cells loaded with EGTA (n = 10, black) or BAPTA (n = 9, gray) in response to a train of ten depolarizing pulses of 50 ms (2 Hz). Note that BAPTA induced a clear reduction of exocytosis during the first pulses. (ii) The bar diagram represents the averaged Ca^2+^ currents during train stimulation. (iii) Synchronous capacitance changes along the train. Synchronous exocytosis is defined as the change in capacitance during each stimulus, and measured in a 50 ms window starting 50 ms after each depolarization minus the mean pre-stimulus capacitance also measured in a 50 ms window. *p<0.05; **p<0.001.

Estimations of IRP size were performed by two methodologies. In the first we applied brief pulse depolarizations (from −80 to +10 mV) of increasing durations (between 5 and 50 ms), and IRP was estimated by the asymptote predicted by the fitting of experimental results to a single exponential (Fig. 1A–ii, black line) [4]. The second methodology is based on the application of two pulses of 10 ms (from −80 to +10 mV, (Fig. 1B–i)) given 300 ms apart [5], [15], [23]. Although the first and second Ca^2+^ currents induced by this protocol were always identical (Fig. 1B–i), the ΔC_m_ exhibited a clear depression between the first and the second pulse (Fig. 1B–ii). This methodology assumes that (i) first and second Ca^2+^ currents induced by this protocol were always identical, and (ii) that no refilling of IRP occurs during the 300 ms interval, and therefore the capacitance increase induced by the second pulse should be significantly depressed with respect to the first one. Upper (B_max_) and lower (B_min_) bounds for this pool size can be calculated according to the equations [5], [15], [24]:
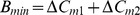


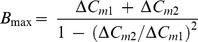
where ΔC_m1_ and ΔC_m2_ represent the capacitance responses to the first and second depolarizations, respectively.

To estimate the RRP we used a paired pulse protocol, but composed by two pulses of 100 ms (from −80 to +10 mV) given 300 ms apart [5], [25].

The capacitance increase caused by individual depolarizations or by each depolarization in paired pulse stimulation was determined as the difference between the mean capacitance measured in a 50 ms window starting 100 ms after the depolarization minus the mean pre-stimulus capacitance also measured in a 50 ms window. The first 100 ms of the capacitance record after depolarization was neglected to avoid non-exocytotic capacitance effects [4], [24].

Trains of depolarizations were composed of 10 pulses, 50 ms each, delivered at 2 Hz frequency. Synchronous exocytosis is defined as the change in capacitance during each stimulus, and measured in a 50 ms window starting 50 ms after each depolarization minus the mean pre-stimulus capacitance also measured in a 50 ms window.

### Fluorescence Measurement of Exocytosis

The styryl dye FM4-64 was used to determine the exocytosis of secretory vesicles in response to long lasting depolarizations. This technique provides a cumulative measurement of exocytosis independently of endocytosis, in contrast to the measurement of capacitance changes which result from a balance between both processes [26]. The imaging setup comprised an inverted fluorescence microscope (Olympus IX81) with a 60X oil immersion objective, a cold CCD camera (Cool Snap HQ_2_, Photometrics, Tucson, AZ) and a personal computer. Illumination was achieved with a 100 Watts mercury lamp, and an epifluorescence filter block containing a 595 nm dichroic mirror, a 560/55 nm band pass excitation filter and a 645/75 nm emission filter. The fluorescence images were acquired using the software Metamorph 8.0 (Molecular Devices Inc. Sunnyvale, California, USA). The standard solution used in these experiments had the following composition (mM): 144 NaCl, 5.6 KCl, 10 Hepes, 1.2 MgCl_2_, 2 CaCl_2_, 10 glucose. The cells were washed in this solution and then exposed to FM4-64 (5 µM) during 15 min. During this period the dye was incorporated into the membrane and fluorescence reached a steady state. Exocytosis was elicited by exchanging the standard solution to one containing 50 mM KCl (in replacement of an equimolar amount of NaCl) and an identical concentration of FM4-64. The magnitude of exocytosis induced by this treatment was estimated by the fractional increased in fluorescence (measured at the end of 3 min high K^+^ stimulation) relative to the preceding basal condition.

#### Generation of synprint expression constructs

A restriction fragment encoding the *Rattus norvegicus* Ca_v_2.2 synprint region was obtained by using the enzymes *Xho*I and *Eco*RI (NEB) to digest a sample of a pTrcHisC subclone of the synprint region [27]. The resulting synprint fragment was run on a 0.8% agarose gel, extracted and purified using a QIAquick Gel Extraction kit (QIAGEN), subcloned into a pIRES2-EGFP vector (BD Biosciences/clonetech) using standard procedures, and sequenced to confirm that the resulting construct correctly encoded the *R. norvegicus* synprint fragment. An EndoFree Plasmid Maxi Kit (QIAGEN) was used to prepare a sample of the construct suitable for transfection into cultured mammalian cells. There is >90% homology between rat and mouse synprint (see Material S1).

#### Materials

Bovine serum albumin, poly-L-lysine, cytosine-1-beta-D-arabinofuranoside, papaine, and the anti-rabbit rhodamine-labeled antibody were obtained from Sigma (St Louis, MO, USA); DMEM, fetal calf serum, gentamicin and penicillin/streptomicin from Gibco (Carlsbad, CA, USA); nitrendipine from Tocris Bioscience (Park Ellsville, MO, SA), ω-agatoxin IVA and the rabbit anti P/Q antibody were obtained from Alomone Labs (Har Hotzvim Hi-Tech Park, Jerusalem, Israel); and FM4-64 from Molecular Probes (Portland, OR).

#### Data analysis and statistics

Images from FM4-64 experiments were quantified with the software Image J (National Institutes of Health, Bethesda, Maryland, USA) by measuring the spatially averaged fluorescence of the whole cell at the equatorial section, and subtracting the background fluorescence (quantified from the surrounding field). Capacitance measurements and Ca^2+^ currents were analyzed with jClamp (Sci Soft, Branford, CT, USA) and Origin (Microcal Sotware Inc, Northhampton, MA) software. Data are expressed as mean values ± standard error. We used a Student’s “t” test for comparisons between two groups of independent data samples, and one way ANOVA for multiple independent data samples.

## Results

### Estimation of IRP in Mouse Chromaffin Cells

The exocytosis of the IRP was classically studied by measuring the synchronous changes in whole cell membrane capacitance (C_m_) in response to brief depolarizations of increasing durations [2], [4], [5], [23]. In agreement with these previous studies, the C_m_ augmentation increased with pulse duration (Fig. 1A), and followed a saturation behavior for pulses ≤50 ms (Fig. 1A–i and Fig. 1A–ii, black circles). This behavior was generally interpreted as the result of the depletion of IRP, and the final asymptote was normally used as an estimation of IRP size [2], [4], [5]. Applying a mono-exponential fit (solid black line) to the experimental data (black circles) represented in Fig. 1A–ii, we estimated an IRP size of 33±1 fF, and a time constant of 15.7±0.8 ms. Depolarization pulses longer than 50 ms induced a delayed second component of exocytosis, which deviated from simple exponential behavior, probably because of the recruitment of vesicles from other pools (Figure 1A–i).

Another strategy previously used to quantify the IRP in chromaffin cells was the application of a paired pulse protocol [5], [15], [24], [25], composed by two square depolarizations of 10 ms given 300 ms apart (see Methods). Figure 1B shows representative recordings of the Ca^2+^ currents (I_Ca2+_) and C_m_ resulting from the application of this paired pulse protocol in control conditions. In agreement with the assumptions of this methodology (see Methods), the I_Ca2+_ activated in response to both depolarizations were identical (see Fig. 1B–i for a single experiment; while the averages were 163±13 and 160±15 pA respectively (n = 14)), and the C_m_ jump induced by the second depolarization (Fig. 1B–ii) was evidently depressed (ΔC_m1_ = 23±3 and ΔC_m2_ = 9±1 fF, n = 14, p<0.001). Capacitance changes induced by the paired pulse protocol were used to calculate a lower (B_min_) and an upper (B_max_) limit for IRP size (see Methods) [5], [15]. The average B_min_ and B_max_ values in control conditions were 32±2 fF and 41±3 fF respectively (Fig. 1C–i, EGTA). Assuming a vesicle capacitance value of 1.3 fF [28], those values are equivalent to 25–32 vesicles. It is important to note that both methodologies gave similar values for the IRP size, as B_min_ and B_max_ bounds include the saturation value obtained from the mono-exponential fitting (Fig. 1A–ii, black circles).

### IRP Exocytosis is Coupled to P/Q-type Calcium Channels

Intracellular Ca^2+^ buffering conditions severely affect the spatial pattern of Ca^2+^ signals [1], [29]. The rapid Ca^2+^ buffer BAPTA, has a 100-fold higher calcium binding rate than EGTA, but a similar Kd (approximately 0.2 µM) [1], [14], [30], [31]. Therefore, BAPTA is capable of binding the incoming Ca^2+^ close to the channel’s mouth, thus reducing the fast secretory component induced by brief depolarization pulses [1], [32]. If IRP vesicles are situated close to the calcium source (approximately 30 nm according to Klingauf and Neher (1997) and Segura et al. (2000)), its secretion should be more sensitive to BAPTA than to EGTA [23]. To evaluate this hypothesis in our mouse chromaffin cell preparation, we analyzed the effect of BAPTA on IRP exocytosis. Both buffers, BAPTA or EGTA respectively, were added to the internal pipette solution at identical concentrations (0.5 mM) in independent experiments. We observed a clear inhibitory effect of BAPTA on the increase of C_m_ provoked by depolarizations shorter than 50 ms (Fig. 1A–ii, gray squares). To release approximately 50% of the IRP in the presence of EGTA, we had to depolarize the cells for 10 ms, whereas equivalent levels of exocytosis in the presence of BAPTA required the application of 40 ms pulses (Fig. 1A–ii, gray dotted line). Furthermore, when we used the paired pulse protocol to estimate IRP, BAPTA treatment significantly reduced B_min_ and B_max_ in comparison with EGTA (p<0.001) (Fig. 1C–i, and for individual experimental examples see Material S2-A), even though calcium current densities under both condition remained similar (21±4 pA/pF vs 25±4 pA/pF, respectively).

Strong stimuli induce prominent Ca^2+^ entry, and consequently provoke the exocytosis of poorly coupled vesicles [2], [23]. At distances relatively far from Ca^2+^ channels (>200 µm) EGTA and BAPTA are expected to buffer Ca^2+^ similarly and therefore to affect exocytosis with comparable efficacy [1]. We tested the effect of BAPTA on the amount of vesicles released by a pair of 100 ms pulses, given 300 ms apart (for a single experiment, see Material S2-B), a stimulation protocol previously used to estimate the lower and upper bounds of the RRP (see Methods) [5], [15]. As expected, in response to this stimulation protocol, cells loaded with BAPTA showed similar values for B_min_, B_max_ (Fig. 1C–ii) and I_Ca2+_ densities compared to cells loaded with EGTA (averaged currents were 19±3 pA/pF vs 23±5 pA/pF, respectively). Another way to provoke the release of poorly coupled vesicles from the RRP is through the application of a train of depolarizations (10 pulses, 50 ms each, delivered at 2 Hz frequency), which leads to significant residual Ca^2+^ accumulation [2]. In the presence of EGTA, the application of such a train induced a cumulative capacitance increase of 150±23 fF (Fig. 1D–i, black line). Note that a very similar capacitance value was obtained in cells loaded with BAPTA at the end of the train (Fig. 1D–i, gray line), and that the current densities along the train were similar in both buffering conditions (Fig. 1D–ii). However, BAPTA provoked a clear decrease in exocytosis during the beginning of the train (Fig. 1D–i, gray line), which was mostly associated with a reduction of the syncronous exocytotic response (see methods and the legend of Fig. 1) during the first four pulses (p<0.05) (Fig. 1D–iii). The greatest effect occurred during the first 50 ms pulse, where most of IRP is released (p<0.001).

**Figure 2:**
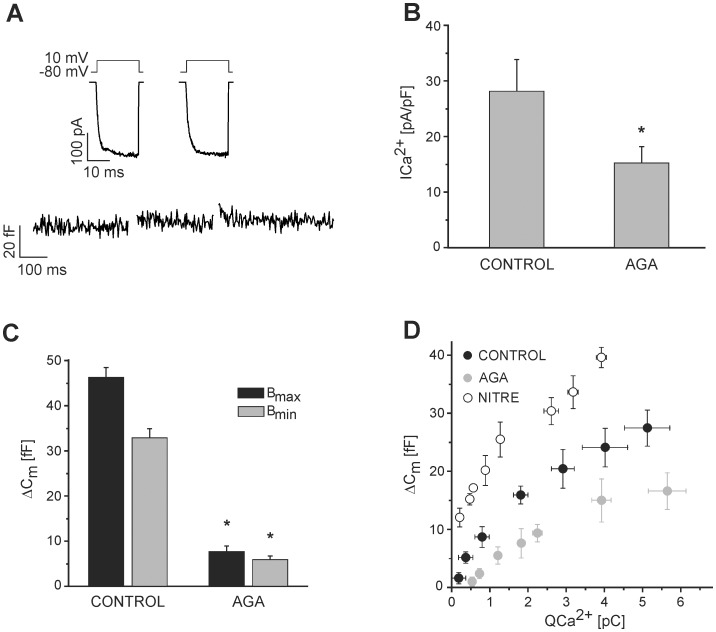
IRP exocytosis is coupled to P/Q calcium channels. A. Representative example of recorded Ca^2+^ currents (top) and membrane capacitance changes (bottom) induced by application of the dual 10 ms pulse protocol in presence of AGA (200 nM) in the external solution. **B.** The bar diagram represents the averaged Ca^2+^ currents in control conditions and in the presence of AGA (n = 8). **C.** The bar diagram summarizes the averaged capacitance changes obtained in response to the application of the dual 10 ms pulse protocol in control conditions and in the presence of AGA (n = 8). The toxin reduced dramatically both B_max_ and B_min_ parameters associated with the exocytosis of the IRP. **D.** Exocytosis, measured as the change in membrane capacitance, in response to short depolarizations (between 5 and 50 ms) was plotted against the Ca^2+^ entry (calculated as the time integral of I_Ca2+_) for cells in control conditions (n = 130), and in presence of NITRE (10 µM) (n = 45) or AGA (n = 45).

The results described above confirm the presence of two components of exocytosis with different functional coupling with Ca^2+^ channels in the chromaffin cell. In a previous study [15], we used specific Ca^2+^ channels blockers and Cav2.1 knockout mice to demonstrate that the exocytosis of IRP is specifically coupled to P/Q-type Ca^2+^ channels in mouse chromaffin cells. Here, we re-evaluated this finding by application of the P/Q channel specific blocker ω-agatoxin-IVA (AGA) (200 nM) on cells stimulated with depolarization pulse protocols specifically intended to release the IRP (Fig. 2). We first analyzed the effect of the toxin on the exocytosis induced by the paired-pulse protocol designed to estimate the IRP (see above). Figure 2A shows an example of the calcium currents and the capacitance changes measured in those conditions. The treatment produced a moderate but significant decrease in calcium current density (p<0.05), and resulted in an 82% reduction of IRP exocytosis (p<0.001) (Figure 2 B and C). This result confirms our previous observation [15], but it does not definitively prove that P/Q-type channels dominate the control of IRP release, as it is well known that exocytosis follows a nonlinear dependence on Ca^2+^ concentration [2], [33]. P/Q plus L-type Ca^2+^ channels account for approximately 100% of the Ca^2+^ current in our chromaffin cell preparation [15] (see also an example of consecutive additions of AGA and nitrendipine (10 µM) on the same cell, in Material S3). Indeed, in the present work, the independent treatment with AGA reduced the I_Ca2+_ to 68±16% and with nitrendipine (NITRE) to 33±9% with respect to controls (see next section, calculated for depolarizations to +10 mV). Therefore, we analyzed the effect of AGA and NITRE on the efficiency of Ca^2+^ entry to induce exocytosis. To perform this analysis, we stimulated the cells with individual depolarization pulses of variable durations (but no longer than 50 ms) and plotted the change in C_m_ vs. I_Ca2+_ integral (QCa^2+^) in cells maintained in control conditions, or in cells treated with AGA, or NITRE (10 µM), respectively (Fig. 2D). When the L-type was the dominant current (i.e. in presence of AGA) there was a clear reduction of the capacitance response in the whole curve in comparison with control conditions. The minimum I_Ca2+_ integral that produced a measurable change in capacitance was right shifted in approximately 0.4 pC with respect to controls. On the other hand, when P/Q-type was the dominant current (i.e. in presence of NITRE) we observed the opposite effect, as the capacitance response was increased in the whole range of I_Ca2+_ integrals. To numerically evaluate the efficiency of Ca^2+^ entry to induce exocytosis in the three conditions considered here, we estimated the I_Ca2+_ integral that induces a capacitance increase of 16 fF (which represents approximately the half of the IRP). On average, a jump in capacitance of this magnitude was provoked by a Ca^2+^ entry of 5.6 pC in experiments with dominant L-type currents (AGA), by a Ca^2+^ entry of 1.9 pC in control conditions, and by a Ca^2+^ entry of 0.7 pC in cells with dominant P/Q-type currents (NITRE). The efficiency of Ca^2+^ entry to induce exocytosis, averaged for all Ca^2+^ entry values in each condition, was also significantly (p<0.05) reduced by AGA (3.5±0.4 fF/pC) and increased by NITRE (18.8±2.8 fF/pC), compared to the value of 8.8±1.4 fF/pC obtained in control cells. This analysis avoids the effect of non-linearity in the exocytosis-I_Ca2+_ relationship [33], confirming the presence of a specific tight coupling between P/Q-type calcium channels and IRP vesicles. Along these lines, we selected from our total data set of paired-pulse experiments for IRP determination in presence of AGA, experiments with Ca^2+^ currents over 20 pA/pF. This yielded an average of 24.2±1.3 pA/pF which was not statistically different from control conditions. In this situation, the estimated IRP was almost identical to the value reported in Fig. 2C in the presence of AGA (Material S4).

### Effect of Synprint on IRP Exocytosis

**Figure 3:**
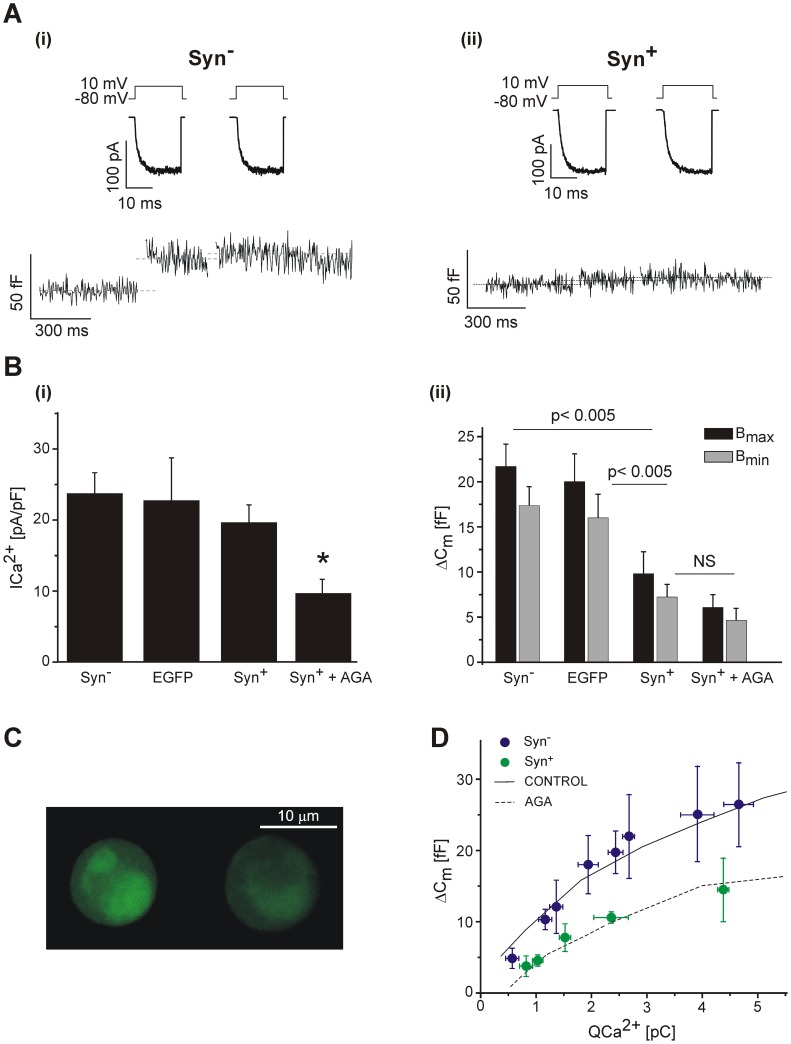
Synprint mediates the coupling of IRP with P/Q-type calcium channels. A: Examples of original records of Ca^2+^ currents (top) and membrane capacitance changes (bottom) in response to the application of the dual 10 ms pulse protocol, in Syn^−^ (i) or Syn^+^ (ii) cells. **B.** (i) Calcium current densities obtained in Syn^−^ (n = 30), EGFP (n = 7), Syn^+^ (n = 14), and Syn^+^ cells treated with 200 nM ω-agatoxin-IVA (Syn^+^+AGA) (n = 10). (ii) Averaged estimations of B_min_ and B_max_ for the IRP, obtained in response to the application of the dual 10 ms pulse protocol under the same conditions mentioned in (i). Please note that while Syn^−^, EGFP and Syn^+^ have almost identical I_Ca2+_ values, a highly significant decrease (p<0.005) in the IRP exocytosis was found between Syn^+^ and the other two groups of experiments. **C.** Confocal images of two chromaffin cells that were positive for EGFP+synprint transfection. **D**. Exocytosis, measured as the change in membrane capacitance in response to short depolarizations (between 5 and 50 ms), was plotted against the Ca^2+^ entry (calculated as the time integral of I_Ca2+_) for Syn^−^ (n = 40) and Syn^+^ cells (n = 30). The plot also represents the data of cells in control conditions (continuous line) or treated with AGA (dashed line) from Fig. 2D. Note that while Syn^−^ followed a similar behavior than control cells, Syn^+^ is superimposed almost perfectly with AGA.

We next examined the molecular basis of the specific coupling between IRP vesicles and P/Q-type Ca^2+^ channels. It has been demonstrated in neurons that the synprint sequence of the α_1_ subunit of P/Q- and N-type Ca^2+^ channels interacts with proteins of the exocytotic machinery [18]. This is thought to contribute to the spatial colocalization of synaptic vesicles with those channels such that Ca^2+^ influx is efficiently coupled to exocytosis [18], [19], [34]. Here, we tested the hypothesis that the tight functional coupling between the IRP exocytosis and the P/Q-type Ca^2+^ current observed in mouse chromaffin cells might be mediated by the P/Q-type channel synprint region. We analyzed this possibility by transfecting mouse chromaffin cells with an IRES plasmid encoding the synprint peptide and EGFP (see methods). We expected that the exogenous free synprint peptide would compete with the endogenous synprint site of the Ca^2+^ channel molecule, thus disrupting the vesicle-channel interaction. We identified the positively transfected cells (Syn^+^) in our cultures by EGFP associated fluorescence (Fig. 3, see also Material S5A). The transfection with synprint did not modify the P/Q-type channel distribution, as assessed with anti-P/Q antibody in fixed and permeabilized chromaffin cells (Material S5B). The values of Ca^2+^ currents and exocytosis measured in Syn^+^ cells were compared with two types of control cells: non fluorescent cells (Syn^−^) chosen from the same dishes than Syn^+^ cells, and EGFP positive cells (EGFP) obtained in independent cultures transfected with an IRES plasmid lacking the synprint sequence.

We applied the double pulse protocol described above to estimate the IRP in Syn^+^, Syn^−^ and EGFP cells. Fig. 3A shows typical examples of individual measurements of I_Ca2+_ and C_m_ in Syn^+^ (i) and Syn^−^ (ii). These examples show that the exocytotic response to double pulse stimulation is markedly reduced in Syn^+^ cells, while I_Ca2+_ was comparable between both situations. On average, I_Ca2+_ was similar for Syn^+^, Syn^−^ and EGFP cells (Fig. 3B–i). In contrast, Syn^+^ cells presented a markedly reduced IRP in comparison to both control conditions (Fig. 3B–ii), (p<0.005). These data suggest that the functional coupling between IRP vesicles and P/Q calcium channels was perturbed by the presence of the exogenous synprint peptide, and consequently IRP exocytosis was inhibited. Consistent with this interpretation, the application of the specific P/Q-type channel blocker AGA on Syn^+^ cells (Syn^+^+AGA) did not significantly alter residual IRP exocytosis measured on Syn^+^ cells (Fig. 3B–ii). We tested also the effect of another cytosolic portion of Ca^2+^ channels, which does not interact with synaptic proteins [35]. We transfected chromaffin cells with the proximal C-terminus of the Cav2.2 channel (residues 1706–1983) incorporated in a pIRES2-EGFP plasmid [36]. This construct did not affect IRP exocytosis in comparison with control measurements obtained from the same cultures (B_max_: 12±3 vs. 13±2, and B_min_: 10±3 vs. 8±1; for C-terminus-1706–1983 (n = 8) and controls (n = 8) respectively). We also tested the effect of synprint transfection on the size of the RRP, evaluated by a paired 100 ms pulse protocol (see methods section, and Ref. [5]). The size of the RRP estimated by this methodology was similar between Syn^−^ (n = 16) and Syn^+^ (n = 20) cells (B_max_ values were 53.9±6.9 and 52.5±8.6 fF; B_min_ values were 40.9±4.1 and 39.1±6.2 fF; and I_ca2+_ values were 16.3±1.9 and 12.3±0.8 fF, respectively).

Next, we analyzed the effect of synprint on the efficiency of Ca^2+^ entry to induce the exocytosis of IRP. The capacitance change provoked by depolarization pulses with durations between 5 and 50 ms was plotted versus the calcium current integral for Syn^+^ and Syn^−^ (Fig. 3D). The exocytosis values obtained on Syn^+^ were reduced in the whole range of I_Ca2+_ integrals relative to Syn^−^. The amount of Ca^2+^ entry required to induce a capacitance increase of 16 fF was 1.8 pC in Syn^−^, but was approximately 5 pC in Syn^+^. Moreover, the average efficiency of Ca^2+^ entry to induce exocytosis was significantly (p<0.001) reduced in Syn^+^ (4.4±0.2 fF/pC) with respect to Syn^−^ (8.0±0.5 fF/pC). Interestingly, while Syn^−^ followed a similar behavior as nontransfected controls (continuous black line, which represents the black filled circles of Fig. 2D), Syn^+^ behaved similarly as cells treated with AGA (dashed black line, which represents the gray circles of Fig. 2D). These results indicate that the blocking of P/Q Ca^2+^ channels or the disruption of P/Q-type channel-vesicle interaction produce similar effects on IRP exocytosis.

**Figure 4:**
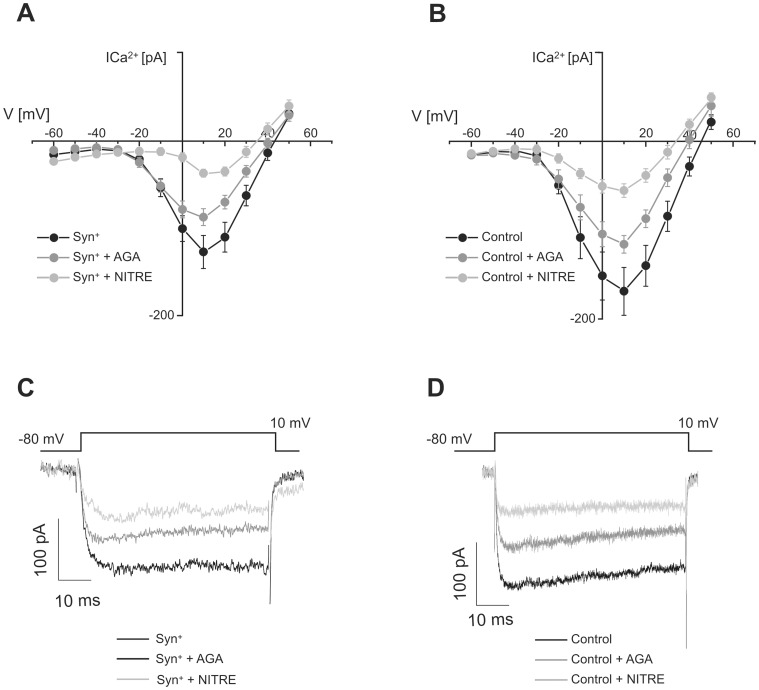
Synprint transfection does not modify the relative contributions of calcium current subtypes. The figures on the top show the calcium current vs. voltage relationships for (**A**) Syn^+^ cells in control conditions (Syn^+^) (n = 9), Syn^+^ cells treated with 200 nM ω-agatoxin-IVA (Syn^+^+AGA) (n = 9), and Syn^+^ cells treated with 10 µM nitrendipine (Syn^+^+NITRE) (n = 7); and for (**B**) nontransfected cells in control conditions (Control) (n = 11), nontransfected cells treated with 200 nM ω-agatoxin-IVA (Control+AGA) (n = 9), and nontransfected cells treated with 10 µM nitrendipine (Control+NITRE) (n = 9). The cells were stimulated with 50 ms square voltage pulses, from a holding potential of −80 mV to the potentials indicated in the abscissas of panels A and B. **C**. Original records of Ca^2+^ currents obtained in response to square depolarizations to +10 mV for the same three conditions detailed in panel A. **D**. Original records of Ca^2+^ currents obtained in response to a square depolarization to +10 mV for the same three conditions detailed in panel B.

In addition to participating in the location of vesicles near the Ca^2+^ channels, the interaction between synprint and SNARE proteins was also implicated in the modulation and targeting of calcium channels to the plasma membrane [34], [37]–[39]. To study the contributions of L- and P/Q-type Ca^2+^ channel to the total I_Ca2+_ in Syn^+^ cells and in non transfected cells we used the specific channel blockers NITRE (10 µM) and AGA (200 nM). The relative contributions of calcium current subtypes to the total current were similar between both preparations. Figure 4A shows the current versus the applied voltage curves obtained in transfected Syn^+^ cells without treatment (Syn^+^), incubated with AGA (Syn^+^+AGA) or with NITRE (Syn^+^+NITRE). AGA significantly reduced the I_Ca2+_ in Syn^+^ cells (to 64%, p<0.05, measured at +10 mV), but a stronger effect was observed with NITRE, which reduced I_Ca2+_ to 41% (p<0.02). Likewise, in control (nontransfected) cells, AGA and NITRE reduced I_Ca2+_ to 68% and 33%, respectively (Fig. 4B). These results confirmed that L-type I_Ca2+_ is the dominant Ca^2+^ current in our preparation (Alvarez et al. 2008), and in addition they showed that synprint transfection do not modify this situation.

### Effect of Synprint on Exocytosis Provoked by Strong Stimulation

We then tested the effect of synprint transfection on chromaffin cell exocytosis induced by strong stimuli. We intended to induce an extensive cytosolic Ca^2+^ increase to trigger the fusion of a big population of vesicles, irrespective of their colocalization with Ca^2+^ channels.

**Figure 5:**
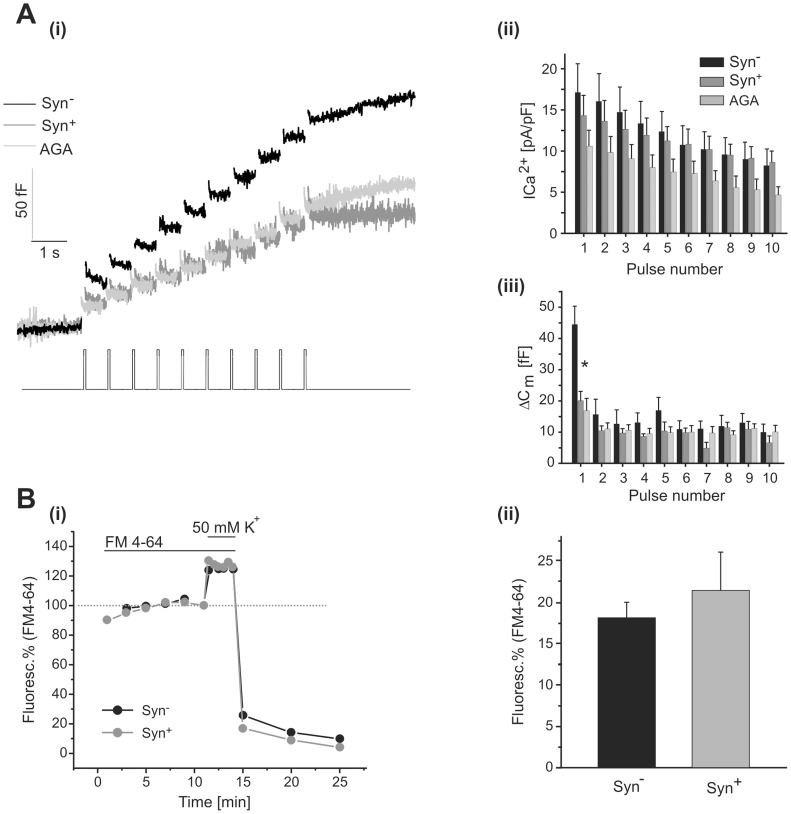
Effects of synprint on exocytosis provoked by strong stimulation. A. (i) Averaged exocytic response of Syn^−^ cells (n = 7, black line), Syn^+^ (n = 6, dark gray line) and normal cells treated with AGA (n = 13, light gray line), in response to trains of ten depolarizing pulses of 50 ms (2 Hz). The bar diagrams in (ii) and (iii) represent the averaged I_Ca2+_ peaks and the synchronous exocytosis elicited at each depolarization in the three conditions described in (i). **B**. The exocytosis was measured as the increase in fluorescence due to incorporation of the fluorophore FM4-64 into the membrane of newly fusing vesicles. (i) Typical experiments performed on a Syn^−^ (n = 10) and a Syn^+^ (n = 9) chromaffin cells. The spatially averaged fluorescence (F.A.U.: fluorescence arbitrary units) of the whole cell was measured at the equatorial cell section, previous subtraction of the background fluorescence (see methods), and normalized (in %) with respect to the value obtained at the end of the FM4-64 incubation period. The cell was stimulated for 3 min. with 50 mM K^+^ solution in presence of the fluorophore. Exocytosis was quantified by the increase in fluorescence above the plateau value established previously the stimulus (dotted line). The stimulation period was terminated by changing the extracellular high K^+^ solution to standard solution without FM4-64 (see methods). (ii) Bar diagram showing the results obtained from the type of experiments represented in (i). The results are expressed as the percentage increase in fluorescence induced by depolarization with respect to previous fluorescence values (after background subtraction). No significant differences were found between these two groups.

First, we examined the response to the application of trains composed by 10 depolarizations (50 ms each, at 2 Hz). In control Syn^−^ cells, this stimulus induced a capacitance increase of 161±20 fF at the end of the train (Fig. 5A–i, black line), which was similar in size to the RRP estimated by other authors [5], [40]. On the other hand, the same stimulation procedure applied in Syn^+^ (dark gray line), or in control cells incubated with AGA (light gray line) provoked a significantly smaller cell capacitance increase (90±16 fF and 98±17 fF, respectively, Fig. 5A–i). It is important to note that approximately 50% of the difference observed between Syn^−^ cells and the two other conditions (AGA and Syn^+^) can be explained by the reduction of the synchronous C_m_ response induced by the first pulse of the train (Fig. 5A–iii). The calcium current densities were not significantly reduced in Syn^+^ cells in comparison with Syn^−^ cells (Fig. 5A–ii), while the application of AGA provoked an average reduction of 46% respect to the control condition (p<0.05). It is interesting to note again (as was also observed in Figure 3D) that synprint transfection produced a similar effect on exocytosis compared to the inhibition of P/Q Ca^2+^ channels by AGA, which fits with the idea that the decrease in exocytosis in Syn^+^ cells is due to the uncoupling of IRP vesicles respect to P/Q-type Ca^2+^ current. These results also make evident that the inhibitory effects of synprint transfection or AGA application are mainly effective on rapid highly synchronous exocytosis during the first pulse of the train, which by definition depends on IRP [2], [4]. On the other hand, most of the remaining exocytosis measured during the train is probably associated with the other vesicles within the RRP, which are not tightly coupled to calcium channels [4], [5]. It is also important to consider that the time length of the train is very close to the refilling time constant of RRP from upstream pools [2], [5].

The results obtained in Syn^+^ cells support the hypothesis that the synprint linker of P/Q-type Ca^2+^ channels is fundamental for maintaining a tight functional coupling between these channels and the vesicles forming the IRP. However, we cannot exclude the possibility that Syn^+^ cells might have suffered a partial collapse in their general competence to release vesicles. To evaluate this alternative we measured the increase in FM4-64 fluorescence (see methods) of Syn^+^ and Syn^−^ in response to the application of 50 mM K^+^ during 3 minutes. This type of protocol releases an important portion of the releasable vesicles contained in chromaffin cells [21]. We used this methodology because it is expected that the exocytosis induced by such a long stimuli would be underestimated by capacitance measurements, as a consequence of simultaneous endocytosis. Fig. 5B–i shows an example of this type of protocol applied on a Syn^−^ and a Syn^+^ cell. The bars of Fig. 5B–ii represent the average increase in fluorescence (expressed as the percentage of the cellular fluorescence before the stimulus) induced by high potassium application. No significant differences were found between these two groups, indicating that the expression of synprint does not affect the general cell competence to release vesicles.

## Discussion

The application of single brief depolarization pulses, which activate VDCCs during a short period, revealed the existence of a small group of vesicles highly coupled to the stimulus, i.e. the IRP [4]. Horrigan and Bookman [4] proposed two alternative hypotheses about the nature of IRP vesicles: (1) The IRP might reflect a population of vesicles (possibly small synaptic-like vesicles) that are functionally distinct from the secretory granules that make up the RRP; (2) IRP+RRP represent a homogeneous population of equally fusion-competent vesicles (i.e. docked and primed) that differ in their proximity to Ca^2+^ channels. Subsequent work clearly favored the second hypothesis. Because the IRP was never observed as a different kinetic component in conventional flash photolysis experiments [5], [40], it is reasonable to predict that the IRP is not a pool of vesicles that is intrinsically faster than the RRP. The application of brief depolarization pulses to deplete the IRP immediately before the flash generated a reduction in the fast component of the exocytotic burst associated with the RRP, and this reduction was similar to the IRP size [5]. Therefore, the IRP is commonly defined as a small group of readily releasable vesicles located in close proximity to VDCCs [5].

The use of specific toxins and pharmacological agents demonstrated that chomaffin cells have a heterogeneous population of VDCCs [8], [41]–[43]. For instance, in mouse chromaffin cells, P/Q-, N- and L-type Ca^2+^ channels may contribute to the I_Ca2+_, but show some variations in their contribution depending on the biological preparation (cell culture or slices) and the patch-clamp configuration used [8], [44], [45]. In the same cell type, some contribution of R-type Ca^2+^ channels to I_Ca2+_ [8], which seems to be more prominent in adrenal gland slices compared to isolated cells [45], was also found. Partial contributions of P/Q-, N- and L-type channels to the total I_Ca2+_ were also demonstrated in bovine [11], [42], [46] and rat [47], [48] chromaffin cells. In the rat, β-adrenergic stimulation recruits low voltage activated T-type Ca^2+^ currents in addition to P/Q-, N- and L-type currents [49], provoking an increase in the cellular secretory response [50]. We observed consistently along several years that L-type and P/Q-type are the dominant Ca^2+^ currents in our chromaffin cell preparation [15].

Our research group [15] obtained strong evidence indicating that IRP vesicles are specifically coupled to P/Q-type Ca^2+^ channels in voltage-clamped mouse chromaffin cells. In that previous study, we showed that IRP was dramatically inhibited by blocking or knocking-out P/Q-type Ca^2+^ channels. However, it is necessary to consider that secretion has a strong nonlinear relationship with Ca^2+^ entry [2], [33]. In the present work we thus analyzed the efficiency of secretion at identical Ca^2+^ current amplitudes mobilized through different Ca^2+^ channels, during the application of stimuli that specifically releases IRP vesicles. We estimated that while 5.6 pC of charge have to flow through L-type channels to release the equivalent of 50% of IRP (16 fF), only 0.7 pC have to enter through P/Q-type channels to provoke the same exocytotic response (Fig. 2D). The ratio between these two current values is 8, thus offering insights into the different exocytotic efficiency of these two Ca^2+^ sources. This scenario suggests the existence of some type of specific physical interaction between IRP vesicles and P/Q-type channels. There is substantial evidence indicating that the synprint linker serves to maintain a close physical coupling between vesicles and P/Q-type or N-type Ca^2+^ channels in synaptic terminals, which enhances the stimulus-secretion response [18], [19]. In this work we tested the hypothesis that synprint helps the functional coupling between IRP associated dense core vesicles and P/Q-type Ca^2+^ channels in chromaffin cells.

The IRP size in this work was evaluated by two methodologies. First, we studied the increase in capacitance provoked by single short depolarizations of variable duration. The exocytotic response to square depolarizations ≤50 ms grew exponentially with the duration of the stimulus [2], [4], giving a saturation value of 33±1 fF, and a time constant (τ) of 15.7±0.8 ms, which corresponds to an exocytotic rate (1/τ) of 64 s^−1^. These kinetic values fall in between the values reported by Horrigan and Bookman [4] in bovine chromaffin cells and Voets et al [5] in mouse adrenal slices. Depolarizations longer than 50 ms induced additional exocytosis that deviated from the exponential behavior (see Fig. 1A–i). This delayed exocytotic component may be associated with RRP vesicles that are not closely coupled with VDCC, and/or with the refilling of mature releasable pools with immature vesicles [5]. As a second methodology to estimate IRP we used the conventional dual-pulse protocol (see Methods, and Fig. 1B and C–i) [5], [23]. We obtained values of 32±2 fF and 41±3 fF for lower and upper IRP limits, respectively, in control conditions. The estimations obtained by both methodologies are consistent with each other, as the asymptote value estimated by the former method falls between B_min_ and B_max_ obtained by the latter analysis. These values are similar to those reported by Voets et al [5] in adrenal slices and by Horrigan and Bookman [4] in bovine cell cultures. If we consider an average value of 1.3 fF for a single vesicle [28], we can conclude that the IRP in our experimental conditions is composed of 25–32 vesicles.

To evaluate if the pool estimated by us is compatible with the basic characteristics that define IRP (i.e. a group of vesicles closely associated with VDCC) we compared the effect of slow vs. fast Ca^2+^ buffers on IRP exocytosis. The rapid exogenous buffer BAPTA has approximately 100 times higher calcium binding rate than EGTA with similar Kd [1], [14], [30], [31], and therefore only the former buffer is expected to affect the exocytosis of vesicles located close to Ca^2+^ channels. Our experiments revealed that BAPTA reduced markedly the efficiency of Ca^2+^ entry to induce IRP exocytosis, in comparison to EGTA (Fig. 1 A and C). On the other hand, no inhibitory effect of BAPTA on exocytosis was observed when longer depolarizations were applied: first, the efficiency of Ca^2+^ entry to induce exocytosis in BAPTA approximates that in EGTA containing solutions when depolarization pulses became longer (Fig. 1A–ii); and second, BAPTA was not effective when a paired 100 ms pulse protocol (to release the whole RRP) was applied. Finally, while the synchronous exocytosis induced by the first pulse of a train was severely affected by BAPTA, this fast buffer had no effect on the total exocytosis at the end of the train (Fig. 1 D). These results are in agreement with a scenario in which there is just a small group of ready releasable vesicles that are highly coupled with VDCCs. How close to the mouth of Ca^2+^ channels are located the vesicles of IRP? We cannot answer this question, but it is possible to estimate a distance range, based on the characteristics of Ca^2+^ buffers. It was estimated that at more than 200 nm from the Ca^2+^ channel the incoming Ca^2+^ is in equilibrium with the buffers [1] and therefore BAPTA and EGTA are expected to produce similar effects. On the other hand, BAPTA presents a length constant, i.e. the average distance a Ca^2+^ ion will diffuse before it is capture by the buffer, of approximately 30 nm [51]. This, together with our results suggests that IRP vesicles are located no more than a few tens of nanometers away from the mouth of the channels.

To study the molecular basis of the functional coupling observed between IRP and P/Q-type Ca^2+^ channels in mouse chromaffin cells we transfected our preparation with a plasmid containing the synprint sequence. It has been previously shown that the synprint peptide that we used to affect IRP exocytosis has two syntaxin interaction sites [52]. We expected that the “free” synprint peptide expressed from that plasmid competed with the synprint site contained within native P/Q-type Ca^2+^ channels for the binding of proteins directly or indirectly associated to vesicles [19]. This would be expected to disrupt the coupling between IRP vesicles and P/Q-type calcium channels, thereby interfering with IRP exocytosis. It is known that the synprint site is present in N- and P/Q-type channels, but it is absent in L-type calcium channels [16], [19], [34], [53], [54]. Because we did not find significant N-type I_Ca2+_ in our preparation [15], it is reasonable to expect that the effect of the free synprint peptide should occur exclusively via P/Q-type Ca^2+^ channels. We found that the efficiency of Ca^2+^ current to promote exocytosis was drastically reduced in Syn^+^ chromaffin cells to a similar level as that obtained when P/Q-type channels were blocked with AGA (Fig. 3D). Additionally, the exocytotic response to the first pulse of a train of depolarizations was reduced in Syn^+^ cells, similarly to the levels seen in the presence of AGA (Fig. 5A). More importantly, the size of IRP exocytosis was markedly reduced in Syn^+^ cells in comparison with both control conditions (Fig. 3B). These results are in agreement with previous findings in synaptic terminals, where synprint helps the establishment of the tight functional coupling between vesicles and Ca^2+^ channels, thus improving the efficiency of synaptic transmission [18], [19]. They are also consistent with the results of Harkins and colleagues [55] in the mouse pheochromocytoma cell line MPC 9/3L, which contains vesicles and many of the proteins involved in vesicle fusion, but which does not express endogenous Ca^2+^ channels. These authors found that MPC 9/3L cells transfected with channels with a deleted synprint site had lower exocytotic efficiency than cells transfected with wild type channels [55]. It should be noted that these authors induced exocytosis through relatively strong stimulation (i.e. trains of 200 ms depolarizations), and thus they did not specifically address the exocytosis of highly coupled vesicles. In summary, although it was shown previously that synprint is important in the establishment of Ca^2+^ channel-vesicle coupling in the mammalian presynapse and in reconstituted neurosecretory systems, to our knowledge we are the first to report that synprint sequence is critical for highly coupled IRP exocytosis in native chromaffin cells.

Although the synprint sequence is also present in N-type Ca^2+^ channels, we showed in a previous study [15] that our chromaffin cell preparation lacks N-type channels and hence our observed effects cannot be attributed to this channel type. Our work does not exclude the possibility that N-type Ca^2+^ channels can contribute to IRP in other chromaffin cell preparations. Moreover, our data do not exclude that Ca^2+^ channels lacking synprint sequence may participate in highly coupled exocytosis in chromaffin cells. Albillos et al [45] found in perforated patch experiments a Ca^2+^ current, apparently mediated by R-type channels, which was a highly efficient trigger of exocytosis.

In addition to synprint, the C-termini of P/Q- and N-type Ca^2+^-channels have also been implicated in targeting Ca^2+^-channels to the synaptic active zone [56]. Bezprozvanny and collaborators demonstrated by “in vitro” and “in vivo” assays a specific association of the cytosolic carboxyl terminus of the long splice variants of N- and Q- type Ca^2+^ channel pore-forming α_1B_ and α_1A_ subunits with the synaptic modular adaptor proteins Mint1-1 and CASK [35], [57]. This molecular association might recruit these Ca^2+^ channels to a macromolecular signaling complex assembled at synaptic junctions. In fact, the synaptic targeting of these Ca^2+^ channels depends on neuronal contacts and synapse formation [35]. Similarly, Südhof and collaborators using yeast two-hybrid screens identified a direct interaction of the central PDZ-domain of the active-zone protein Rim with the C-termini of presynaptic N- and P/Q-type Ca^2+^-channels [58]. The knockout of Rim strongly reduced neurotransmitter release in hippocampal neurons and in the calyx of Held, revealing a reduction in the size of the readily releasable pool, and a decrease in the presynaptic localization of Ca^2+^-channels [58], [59]. The addition of Rim1 to permeabilized chromaffin cells increased secretion apparently through a mechanism involving Rab3a and/or a 14-3-3 protein [60], [61]. In addition there is data suggesting that the disruption of Mint1-Munc18-1 binding decreases secretion in chromaffin cells [62]. However there is not to our knowledge any data that demonstrate a role of CASK, Rim or Mint1 in channel localization or channel-vesicle coupling in chromaffin cells.

From our results we can also conclude that massive exocytosis provoked by sustained stimuli is not significantly affected by synprint associated vesicle-channel interactions (Fig. 5B). This is reasonable, because prolonged depolarizations provoke global Ca^2+^ increases in the cytosol, which are expected to affect vesicle exocytosis independently of their localization respect to Ca^2+^ sources [2], [29].

In conclusion, our results strongly suggest that synprint is a crucial factor for the establishment of the functional coupling between IRP vesicles and P/Q-type Ca^2+^ channels in mouse chromafin cells. Therefore, synprint appears to be essential for highly synchronized secretion in chromaffin cells, like in the presynapse [17], [19].

## Supporting Information

Material S1 Rat versus mouse synprint homology analysis. Based on the comparison, these segments of the synprint region are 93% identical and 94.7% similar between rat and mouse, with eight gaps in alignment due to the rat synprint isoform in question being longer.
(PDF)

Material S2 **A.** Examples of original capacitance records obtained in response to the application of a 10 ms dual pulse protocol for the estimation of the IRP in cells dialyzed with (i) 0.5 mM EGTA or (ii) 0.5 mM BAPTA. **B.** Original capacitance records obtained in response to the application of a 100 ms dual pulse protocol for the estimation of the RRP in cells dialyzed with (i) 0.5 mM EGTA and (ii) 0.5 mM BAPTA.
(PDF)

Material S3 Examples of I_Ca2+_ induced by 50 ms depolarizations (from −80 to +10 mV) obtained on the same cell in control conditions (black), and with consecutive additions of 200 nM AGA (gray), and AGA +10 µM Nitre (light gray).
(PDF)

Material S4 **A.** The bar diagram compares the averaged Ca^2+^ currents densities in control conditions and in the presence of AGA (replications of Fig. 2B) with a group of experiments with high Ca^2+^ current densities in presence of AGA (we selected 3 cells from the experiments represented in Fig. 2B and C, and added 4 new cells, all with currents higher than 20 pA/pF (n = 7)). The currents were induced by application of a 10 ms square depolarization (the first of the pair). The Ca^2+^ current density obtained in AGA with high I_Ca2+_ was not different than the control. **B.** The IRP size estimated by the dual pulse protocol was markedly smaller in AGA with high I_Ca2+_ than in control conditions (p<0.001), and almost identical to the values obtained for the regular population of experiments performed with AGA. Control and AGA represent the same experiments shown in Fig. 2B and C.
(PDF)

Material S5 **A.** Examples of DIC Nomarsky images and associated confocal EGFP fluorescence images of two fixed chromaffin cells transfected with the synprint-pIRES2-EGFP plasmid. **B.** Examples of cellular P/Q-type channel distribution for control and Syn+ cells. The cells were fixed in 2% paraformaldehyde and permeabilized with 0.5% Tween 20. Subsequently, the cells were incubated overnight with a rabbit anti P/Q antibody (1∶200), and an anti rabbit second antibody labeled with rhodamine (1∶1000) was applied. The images were obtained in an Olympus FV-300 confocal microscope with a 60× (1.4) oil immersion objective.
(PDF)

## References

[1] Neher E (1998) Vesicle pools and Ca^2+^ microdomains: new tools for understanding their roles in neurotransmitter release. Neuron 20: 389–399. 9539117 10.1016/s0896-6273(00)80983-6

[2] Marengo FD (2005) Calcium gradients and exocytosis in bovine adrenal chromaffin cells. Cell Calcium 38: 87–99. 16076487 10.1016/j.ceca.2005.06.006

[3] Robinson IM, Finnegan JM, Monck JR, Wightman RM, Fernandez JM (1995) Colocalization of calcium entry and exocytotic release sites in adrenal chromaffin cells. Proc Natl Acad Sci USA 92: 2474–2478. 7708668 10.1073/pnas.92.7.2474 PMC42240

[4] Horrigan FT, Bookman RJ (1994) Releasable pools and the kinetics of exocytosis in adrenal chromaffin cells. Neuron 13: 1119–1129. 7946349 10.1016/0896-6273(94)90050-7

[5] Voets T, Neher E, Moser T (1999) Mechanisms underlying phasic and sustained secretion in chromaffin cells from mouse adrenal slices. Neuron 23: 607–615. 10433271 10.1016/s0896-6273(00)80812-0

[6] Alvarez YD, Marengo FD (2011) The immediately releasable vesicle pool: highly coupled secretion in chromaffin and other neuroendocrine cells. J Neurochem 116: 155–163. 21073467 10.1111/j.1471-4159.2010.07108.x

[7] Klingauf J, Neher E (1997) Modeling buffered Ca2+ diffusion near the membrane: implications for secretion in neuroendocrine cells. Biophys J 72: 674–690. 9017195 10.1016/s0006-3495(97)78704-6 PMC1185593

[8] Aldea M, Jun K, Shin HS, Andres-Mateos E, Solis-Garrido LM, et al (2002) A perforated patch-clamp study of calcium currents and exocytosis in chromaffin cells of wild-type and alpha(1A) knockout mice. J Neurochem 81: 911–921. 12065603 10.1046/j.1471-4159.2002.00845.x

[9] Engisch KL, Nowycky MC (1996) Calcium dependence of large dense-cored vesicle exocytosis evoked by calcium influx in bovine adrenal chromaffin cells. J Neurosci 16: 1359–1369. 8778287 10.1523/JNEUROSCI.16-04-01359.1996 PMC6578563

[10] Chow RH, Klingauf J, Neher E (1994) Time course of Ca^2+^ concentration triggering exocytosis in neuroendocrine cells. Proc Natl Acad Sci U S A 91: 12765–12769. 7809118 10.1073/pnas.91.26.12765 PMC45520

[11] Lara B, Gandia L, Martinez-Sierra R, Torres A, Garcia AG (1998) Q-type Ca^2+^ channels are located closer to secretory sites than L-type channels: functional evidence in chromaffin cells. Pflugers Arch 435: 472–478. 9446693 10.1007/s004240050541

[12] Wykes RC, Bauer CS, Khan SU, Weiss JL, Seward EP (2007) Differential regulation of endogenous N- and P/Q-type Ca^2+^ channel inactivation by Ca^2+^/calmodulin impacts on their ability to support exocytosis in chromaffin cells. J Neurosci 27: 5236–5248. 17494710 10.1523/JNEUROSCI.3545-06.2007 PMC6672387

[13] Elhamdani A, Zhou Z, Artalejo CR (1998) Timing of dense-core vesicle exocytosis depends on the facilitation L-type Ca channel in adrenal chromaffin cells. J Neurosci 18: 6230–6240. 9698316 10.1523/JNEUROSCI.18-16-06230.1998 PMC6793173

[14] Segura J, Gil A, Soria B (2000) Modeling study of exocytosis in neuroendocrine cells: influence of the geometrical parameters. Biophys J 79: 1771–1786. 11023885 10.1016/S0006-3495(00)76429-0 PMC1301071

[15] Alvarez YD, Ibanez LI, Uchitel OD, Marengo FD (2008) P/Q Ca^2+^ channels are functionally coupled to exocytosis of the immediately releasable pool in mouse chromaffin cells. Cell Calcium 43: 155–164. 17561253 10.1016/j.ceca.2007.04.014

[16] Rettig J, Sheng ZH, Kim DK, Hodson CD, Snutch TP, et al (1996) Isoform-specific interaction of the alpha1A subunits of brain Ca2+ channels with the presynaptic proteins syntaxin and SNAP-25. Proc Natl Acad Sci U S A 93: 7363–7368. 8692999 10.1073/pnas.93.14.7363 PMC38990

[17] Zamponi GW (2003) Regulation of presynaptic calcium channels by synaptic proteins. J Pharmacol Sci 92: 79–83. 12832834 10.1254/jphs.92.79

[18] Catterall WA (1999) Interactions of presynaptic Ca2+ channels and snare proteins in neurotransmitter release. Ann N Y Acad Sci 868: 144–159. 10414292 10.1111/j.1749-6632.1999.tb11284.x

[19] Mochida S, Sheng ZH, Baker C, Kobayashi H, Catterall WA (1996) Inhibition of neurotransmission by peptides containing the synaptic protein interaction site of N-type Ca2+ channels. Neuron 17: 781–788. 8893034 10.1016/s0896-6273(00)80209-3

[20] Andres-Mateos E, Renart J, Cruces J, Solis-Garrido LM, Serantes R, et al (2005) Dynamic association of the Ca2+ channel alpha1A subunit and SNAP-25 in round or neurite-emitting chromaffin cells. Eur J Neurosci 22: 2187–2198. 16262657 10.1111/j.1460-9568.2005.04385.x

[21] Perez Bay AE, Ibanez LI, Marengo FD (2007) Rapid recovery of releasable vesicles and formation of nonreleasable endosomes follow intense exocytosis in chromaffin cells. Am J Physiol Cell Physiol 293: C1509–C1522. 17686997 10.1152/ajpcell.00632.2006

[22] Fidler N, Fernandez JM (1989) Phase tracking: an improved phase detection technique for cell membrane capacitance measurements. Biophys J 56: 1153–1162. 2611329 10.1016/S0006-3495(89)82762-6 PMC1280618

[23] Moser T, Neher E (1997) Rapid exocytosis in single chromaffin cells recorded from mouse adrenal slices. J Neurosci 17: 2314–2323. 9065492 10.1523/JNEUROSCI.17-07-02314.1997 PMC6573505

[24] Moser T, Neher E (1997) Rapid exocytosis in single chromaffin cells recorded from mouse adrenal slices. J Neurosci 17: 2314–2323. 9065492 10.1523/JNEUROSCI.17-07-02314.1997 PMC6573505

[25] Gillis KD, Mossner R, Neher E (1996) Protein kinase C enhances exocytosis from chromaffin cells by increasing the size of the readily releasable pool of secretory granules. Neuron 16: 1209–1220. 8663997 10.1016/s0896-6273(00)80147-6

[26] Smith CB, Betz WJ (1996) Simultaneous independent measurement of endocytosis and exocytosis. Nature 380: 531–534. 8606773 10.1038/380531a0

[27] Jarvis SE, Magga JM, Beedle AM, Braun JE, Zamponi GW (2000) G protein modulation of N-type calcium channels is facilitated by physical interactions between syntaxin 1A and Gbetagamma. J Biol Chem 275: 6388–6394. 10692440 10.1074/jbc.275.9.6388

[28] Moser T, Neher E (1997) Estimation of mean exocytic vesicle capacitance in mouse adrenal chromaffin cells. Proc Natl Acad Sci U S A 94: 6735–6740. 9192634 10.1073/pnas.94.13.6735 PMC21227

[29] Marengo FD, Monck JR (2000) Development and dissipation of Ca(2+) gradients in adrenal chromaffin cells. Biophys J 79: 1800–1820. 11023887 10.1016/S0006-3495(00)76431-9 PMC1301073

[30] Smith PD, Liesegang GW, Berger RL, Czerlinski G, Podolsky RJ (1984) A stopped-flow investigation of calcium ion binding by ethylene glycol bis(beta-aminoethyl ether)-N,N’-tetraacetic acid. Anal Biochem 143: 188–195. 6442108 10.1016/0003-2697(84)90575-x

[31] Tsien RY (1980) New calcium indicators and buffers with high selectivity against magnesium and protons: design, synthesis, and properties of prototype structures. Biochemistry 19: 2396–2404. 6770893 10.1021/bi00552a018

[32] Adler EM, Augustine GJ, Duffy SN, Charlton MP (1991) Alien intracellular calcium chelators attenuate neurotransmitter release at the squid giant synapse. J Neurosci 11: 1496–1507. 1675264 10.1523/JNEUROSCI.11-06-01496.1991 PMC6575403

[33] Dodge FA Jr, Rahamimoff R (1967) Cooperative action a calcium ions in transmitter release at the neuromuscular junction. J Physiol 193: 419–432. 6065887 10.1113/jphysiol.1967.sp008367 PMC1365607

[34] Mochida S, Westenbroek RE, Yokoyama CT, Zhong H, Myers SJ, et al (2003) Requirement for the synaptic protein interaction site for reconstitution of synaptic transmission by P/Q-type calcium channels. Proc Natl Acad Sci U S A 100: 2819–2824. 12601156 10.1073/pnas.262787699 PMC151424

[35] Maximov A, Bezprozvanny I (2002) Synaptic targeting of N-type calcium channels in hippocampal neurons. J Neurosci 22: 6939–6952. 12177192 10.1523/JNEUROSCI.22-16-06939.2002 PMC3307533

[36] Beedle AM, McRory JE, Poirot O, Doering CJ, Altier C, et al (2004) Agonist-independent modulation of N-type calcium channels by ORL1 receptors. Nat Neurosci 7: 118–125. 14730309 10.1038/nn1180

[37] Szabo Z, Obermair GJ, Cooper CB, Zamponi GW, Flucher BE (2006) Role of the synprint site in presynaptic targeting of the calcium channel CaV2.2 in hippocampal neurons. Eur J Neurosci 24: 709–718. 16930401 10.1111/j.1460-9568.2006.04947.x

[38] Jarvis SE, Zamponi GW (2005) Masters or slaves? Vesicle release machinery and the regulation of presynaptic calcium channels. Cell Calcium 37: 483–488. 15820397 10.1016/j.ceca.2005.01.017

[39] Zhong H, Yokoyama CT, Scheuer T, Catterall WA (1999) Reciprocal regulation of P/Q-type Ca2+ channels by SNAP-25, syntaxin and synaptotagmin. Nat Neurosci 2: 939–941. 10526329 10.1038/14721

[40] Ashery U, Varoqueaux F, Voets T, Betz A, Thakur P, et al (2000) Munc13–1 acts as a priming factor for large dense-core vesicles in bovine chromaffin cells. EMBO J 19: 3586–3596. 10899113 10.1093/emboj/19.14.3586 PMC313963

[41] Santana F, Michelena P, Jaen R, Garcia AG, Borges R (1999) Calcium channel subtypes and exocytosis in chromaffin cells: a different view from the intact rat adrenal. Naunyn Schmiedebergs Arch Pharmacol 360: 33–37. 10463331 10.1007/s002109900041

[42] Artalejo CR, Adams ME, Fox AP (1994) Three types of Ca^2+^ channel trigger secretion with different efficacies in chromaffin cells. Nature 367: 72–76. 8107778 10.1038/367072a0

[43] Garcia AG, Garcia-de-Diego AM, Gandia L, Borges R, Garcia-Sancho J (2006) Calcium signaling and exocytosis in adrenal chromaffin cells. Physiol Rev 86: 1093–1131. 17015485 10.1152/physrev.00039.2005

[44] Hernandez-Guijo JM, de Pascual R, Garcia AG, Gandia L (1998) Separation of calcium channel current components in mouse chromaffin cells superfused with low- and high-barium solutions. Pflugers Arch 436: 75–82. 9560449 10.1007/s004240050606

[45] Albillos A, Neher E, Moser T (2000) R-Type Ca^2+^ channels are coupled to the rapid component of secretion in mouse adrenal slice chromaffin cells. J Neurosci 20: 8323–8330. 11069939 10.1523/JNEUROSCI.20-22-08323.2000 PMC6773200

[46] Lomax RB, Michelena P, Nunez L, Garcia-Sancho J, Garcia AG, et al (1997) Different contributions of L- and Q-type Ca2+ channels to Ca2+ signals and secretion in chromaffin cell subtypes. Am J Physiol 272: C476–C484. 9124290 10.1152/ajpcell.1997.272.2.C476

[47] Gandia L, Borges R, Albillos A, Garcia AG (1995) Multiple calcium channel subtypes in isolated rat chromaffin cells. Pflugers Arch 430: 55–63. 7545281 10.1007/BF00373839

[48] Prakriya M, Lingle CJ (1999) BK channel activation by brief depolarizations requires Ca2+ influx through L- and Q-type Ca2+ channels in rat chromaffin cells. J Neurophysiol 81: 2267–2278. 10322065 10.1152/jn.1999.81.5.2267

[49] Novara M, Baldelli P, Cavallari D, Carabelli V, Giancippoli A, et al (2004) Exposure to cAMP and beta-adrenergic stimulation recruits Ca(V)3 T-type channels in rat chromaffin cells through Epac cAMP-receptor proteins. J Physiol 558: 433–449. 15133061 10.1113/jphysiol.2004.061184 PMC1664977

[50] Giancippoli A, Novara M, de Luca A, Baldelli P, Marcantoni A, et al (2006) Low-threshold exocytosis induced by cAMP-recruited CaV3.2 (alpha1H) channels in rat chromaffin cells. Biophys J 90: 1830–1841. 16361341 10.1529/biophysj.105.071647 PMC1367332

[51] Naraghi M, Neher E (1997) Linearized buffered Ca^2+^ diffusion in microdomains and its implications for calculation of [Ca^2+^] at the mouth of a calcium channel. J Neurosci 17: 6961–6973. 9278532 10.1523/JNEUROSCI.17-18-06961.1997 PMC6573285

[52] Davies JN, Jarvis SE, Zamponi GW (2011) Bipartite syntaxin 1A interactions mediate CaV2.2 calcium channel regulation. Biochem. Biophys. Res. Commun. 411: 562–568. 10.1016/j.bbrc.2011.06.185 21763275

[53] Sheng ZH, Rettig J, Takahashi M, Catterall WA (1994) Identification of a syntaxin-binding site on N-type calcium channels. Neuron 13: 1303–1313. 7993624 10.1016/0896-6273(94)90417-0

[54] Spafford JD, Zamponi GW (2003) Functional interactions between presynaptic calcium channels and the neurotransmitter release machinery. Curr Opin Neurobiol 13: 308–314. 12850215 10.1016/s0959-4388(03)00061-8

[55] Harkins AB, Cahill AL, Powers JF, Tischler AS, Fox AP (2004) Deletion of the synaptic protein interaction site of the N-type (CaV2.2) calcium channel inhibits secretion in mouse pheochromocytoma cells. Proc Natl Acad Sci U S A 101: 15219–15224. 15471993 10.1073/pnas.0401001101 PMC524046

[56] Catterall WA, Perez-Reyes E, Snutch TP, Striessnig J (2005) International Union of Pharmacology. XLVIII. Nomenclature and structure-function relationships of voltage-gated calcium channels. Pharmacol Rev 57: 411–425. 16382099 10.1124/pr.57.4.5

[57] Maximov A, Sudhof TC, Bezprozvanny I (1999) Association of Neuronal Calcium Channels with Modular Adaptor Proteins. J Biol Chem 274: 24453–24456. 10455105 10.1074/jbc.274.35.24453

[58] Kaeser PS, Deng L, Wang Y, Dulubova I, Liu X, et al (2011) RIM proteins tether Ca2+-channels to presynaptic active zones via a direct PDZ-domain interaction. Cell 144: 282–295. 21241895 10.1016/j.cell.2010.12.029 PMC3063406

[59] Han Y, Kaeser PS, Sudhof TC, Schneggenburger R (2011) RIM determines Ca2+ channel density and vesicle docking at the presynaptic active zone. Neuron 69: 304–316. 21262468 10.1016/j.neuron.2010.12.014 PMC3259453

[60] Sun L, Bittner MA, Holz RW (2003) Rim, a component of the presynaptic active zone and modulator of exocytosis, binds 14-3-3 through its N terminus. J Biol Chem 278: 38301–38309. 12871946 10.1074/jbc.M212801200

[61] Sun L, Bittner MA, Holz RW (2002) Rim and exocytosis: Rab3a-binding and secretion-enhancing domains are separate and function independently. Ann N Y Acad Sci 971: 244–247. 12438124 10.1111/j.1749-6632.2002.tb04468.x

[62] Graham ME, Prescott GR, Johnson JR, Jones M; Walmesley A, et al (2011) Structure-Function Study of Mammalian Munc18–1 and C. elegans UNC-18 Implicates Domain 3b in the Regulation of Exocytosis. PLoS One 6: 1–12. 10.1371/journal.pone.0017999 PMC3061876 21445306

