# Supplementary material for: The Immediately Releasable Pool of Mouse Chromaffin Cell Vesicles Is Coupled to P/Q-Type Calcium Channels via the Synaptic Protein Interaction Site
Source: PLoS One. 2013 Jan 30;8(1):e54846. doi: 10.1371/journal.pone.0054846 (PMC3559834; doi:10.1371/journal.pone.0054846)
Supplement: Material S1 — Rat versus mouse synprint homology analysis. Based on the comparison, these segments of the synprint region are 93% identical and 94.7% similar between rat and mouse, with eight gaps in alignment due to the rat synprint isoform in question being longer. (PDF) [file pone.0054846.s001.pdf]

Supplemental Material 1: rat vs. mouse synprint homology analysis

>IRES-eGFP::Rn synprint subclone (Jarvis & Tedford)
ELTKDEEEMEEAANQKLALQKAKEVAEVSPMSAANISIAARQQNSAKARSVWEQRASQL
RLQNLRASCEALYSEMDPEERLRYASTRHVRPDMKTHMDRPLVVEPGRDGLRGPAGNKS
KPEGTEATEGADPPRRHHRHRDRDKTSASTPAGGEQDRTDCPKAESTETGAREERARPR
RSHSKEAPGADTQVRCERSRRHHRRGSPEEATEREPRRHRAHRHAQDSSKEGKEGTAPV
LVPKGERRAR

BLAST identified records of two mouse mRNAs with high identity to the above (translated fragments of the sequences and corresponding accession numbers shown below):

>M. musculus mRNA, genbank Accession #:BU056303.1
ELTKDEEEMEEAANQKLALQKAKEV

> M. musculus mRNA, genbank Accession #:CF744357.1
AEVSPMSAANISIAARQQNSAKARSVWEQRASQLRLQNLRASCEALYSEMDPEERLRYA
STRHVRPDMKTHMDRPLVVEPGRDGLRGPVGSKSKPEGTEATESADLPRRHHRHRDRDK
TSATAPAGGEQDRTESTETGAREERARPRRSHSKETPGADTQVRCERSRRHHRRGSPEE
ATEREPRRHRAHRHAQDSSKEGTAPVLVPKGERRAR

>hybrid Mm synprint sequence (used in alignment below)
ELTKDEEEMEEAANQKLALQKAKEVAEVSPMSAANISIAARQQNSAKARSVWEQRASQL
RLQNLRASCEALYSEMDPEERLRYASTRHVRPDMKTHMDRPLVVEPGRDGLRGPVGSKS
KPEGTEATESADLPRRHHRHRDRDKTSATAPAGGEQDRTESTETGAREERARPRRSHSK
ETPGADTQVRCERSRRHHRRGSPEEATEREPRRHRAHRHAQDSSKEGTAPVLVPKGERR
AR

```
Rn synprint  ELTKDEEEMEEAANQKLALQKAKEVAEVSPMSAANISIAARQQNSAKARS  50
Mm synprint  ELTKDEEEMEEAANQKLALQKAKEVAEVSPMSAANISIAARQQNSAKARS  50

Rn synprint  VWEQRASQLRLQNLRASCEALYSEMDPEERLRYASTRHVRPDMKTHMDRP  100
Mm synprint  VWEQRASQLRLQNLRASCEALYSEMDPEERLRYASTRHVRPDMKTHMDRP  100

Rn synprint  LVVEPGRDGLRGPAGNKSKPEGTEATEGADPPRRHHRHRDRDKTSASTPA  150
Mm synprint  LVVEPGRDGLRGPVGSKSKPEGTEATESADLPRRHHRHRDRDKTSATAPA  150

Rn synprint  GGEQDRTDCPKAESTETGAREERARPRRSHSKEAPGADTQVRCERSRRHH  200
Mm synprint  GGEQDRT-----ESTETGAREERARPRRSHSKETPGADTQVRCERSRRHH  195

Rn synprint  RRGSPEEATEREPRRHRAHRHAQDSSKEGKEGTAPVLVPKGERRAR  246
Mm synprint  RRGSPEEATEREPRRHRAHRHAQDSSKEG---TAPVLVPKGERRAR  238
```

Based on the above and results of BLAST alignments, these segments of the synprint region are 93% identical and 94.7% similar between rat and mouse, with eight gaps in alignment due to the rat synprint isoform in question being longer.
