# Supplementary figures and images for: The Immediately Releasable Pool of Mouse Chromaffin Cell Vesicles Is Coupled to P/Q-Type Calcium Channels via the Synaptic Protein Interaction Site
Source: PLoS One. 2013 Jan 30;8(1):e54846. doi: 10.1371/journal.pone.0054846 (PMC3559834; doi:10.1371/journal.pone.0054846)

## Supplemental material 2

### A IRP

#### (i) EGTA

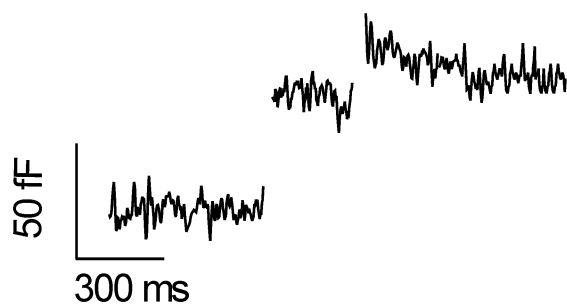

#### (ii) BAPTA

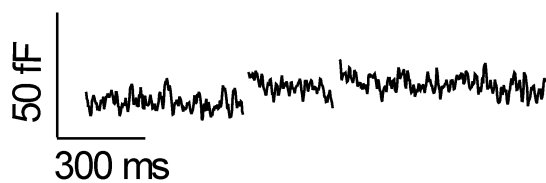

### B RRP

#### (i) EGTA

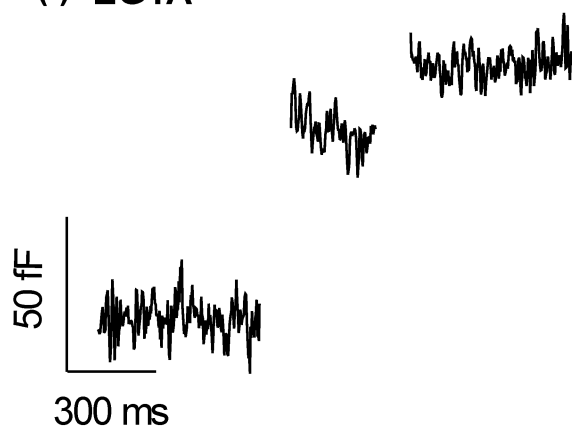

#### (ii) BAPTA

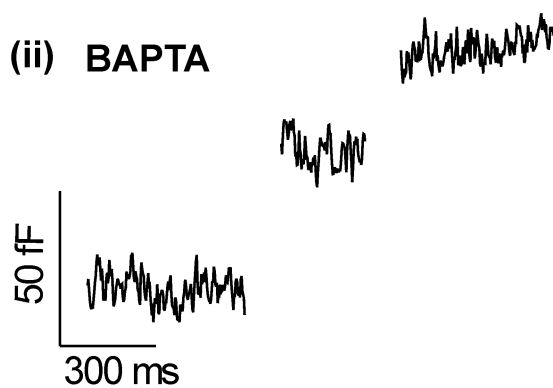

Supplement: Material S2 — A. Examples of original capacitance records obtained in response to the application of a 10 ms dual pulse protocol for the estimation of the IRP in cells dialyzed with (i) 0.5 mM EGTA or (ii) 0.5 mM BAPTA. B. Original capacitance records obtained in response to the application of a 100 ms dual pulse protocol for the estimation of the RRP in cells dialyzed with (i) 0.5 mM EGTA and (ii) 0.5 mM BAPTA. (PDF) [file pone.0054846.s002.pdf]

### Supplemental Material 3

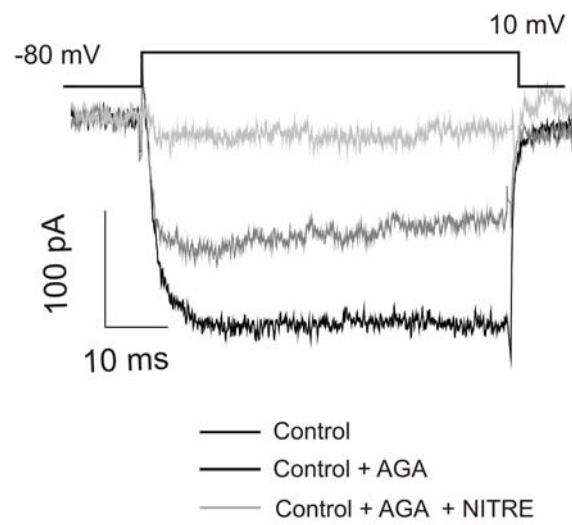

Supplement: Material S3 — Examples of ICa2+ induced by 50 ms depolarizations (from −80 to +10 mV) obtained on the same cell in control conditions (black), and with consecutive additions of 200 nM AGA (gray), and AGA +10 µM Nitre (light gray). (PDF) [file pone.0054846.s003.pdf]

Supplemental material 4

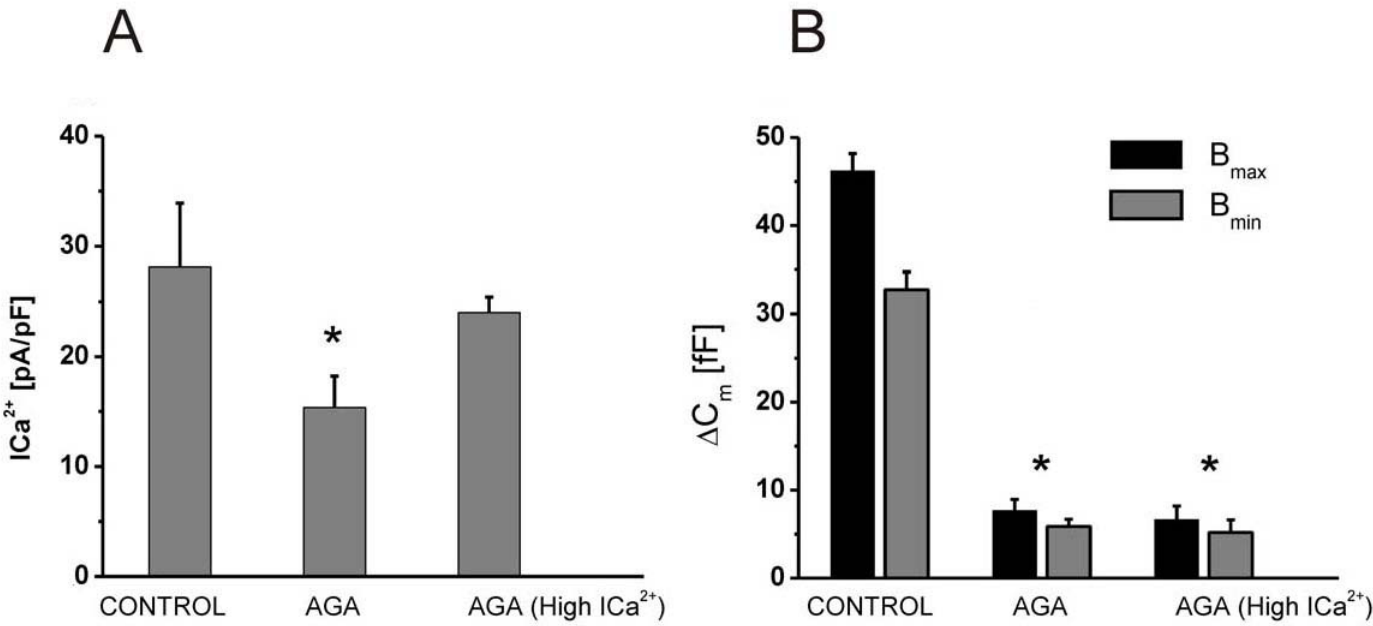

Supplement: Material S4 — A. The bar diagram compares the averaged Ca2+ currents densities in control conditions and in the presence of AGA (replications of Fig. 2B) with a group of experiments with high Ca2+ current densities in presence of AGA (we selected 3 cells from the experiments represented in Fig. 2B and C, and added 4 new cells, all with currents higher than 20 pA/pF (n = 7)). The currents were induced by application of a 10 ms square depolarization (the first of the pair). The Ca2+ current density obtained in AGA with high ICa2+ was not different than the control. B. The IRP size estimated by the dual pulse protocol was markedly smaller in AGA with high ICa2+ than in control conditions (p<0.001), and almost identical to the values obtained for the regular population of experiments performed with AGA. Control and AGA represent the same experiments shown in Fig. 2B and C. (PDF) [file pone.0054846.s004.pdf]

## Supplemental material 5

A

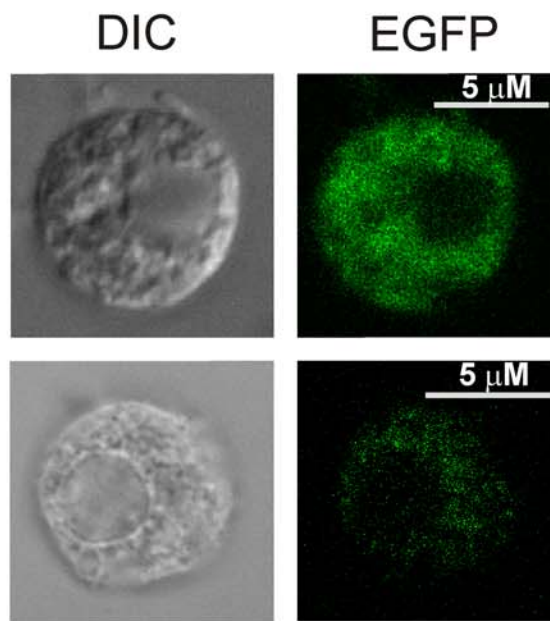

B

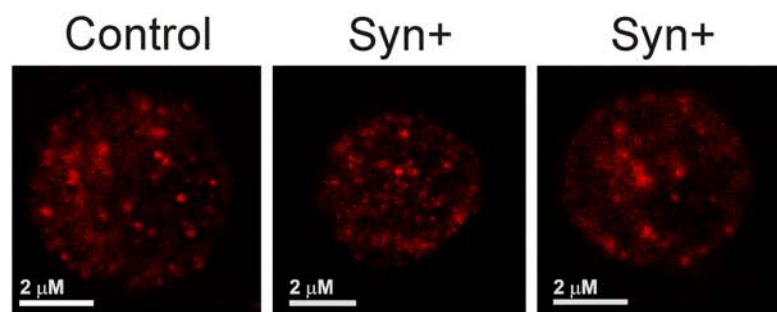

Supplement: Material S5 — A. Examples of DIC Nomarsky images and associated confocal EGFP fluorescence images of two fixed chromaffin cells transfected with the synprint-pIRES2-EGFP plasmid. B. Examples of cellular P/Q-type channel distribution for control and Syn+ cells. The cells were fixed in 2% paraformaldehyde and permeabilized with 0.5% Tween 20. Subsequently, the cells were incubated overnight with a rabbit anti P/Q antibody (1∶200), and an anti rabbit second antibody labeled with rhodamine (1∶1000) was applied. The images were obtained in an Olympus FV-300 confocal microscope with a 60× (1.4) oil immersion objective. (PDF) [file pone.0054846.s005.pdf]
